# The Atlanta Society of Medicine

**Published:** 1886-06-20

**Authors:** J. P. Logan


### EDITORIALS AND MISCELLANEOUS.

### THE ATLANTA SOCIETY OF MEDICINE

gave their first annual banquet last night at Pause's Restaurant. Altogether it was a grand affair, and we doubt whether a more sumptuous entertainment was ever spread before the medical profession in this city. Dr. Campbell, of Augusta, and Dr. Battey, of Rome, were both present on the occasion and responded pleasantly to toasts that were tendered to them. We state this briefly for want of space and time, as we are about going to press, but may refer to this event again in our next issue.

*Dr. R. C. Word, Editor Southern Medical Record:*

DEAR SIR—Please allow me to correct the report, in the last number of the RECORD, of the proceedings of the Georgia Medical Association, so far as it refers to the paper read by myself before that body. The subject of that paper was "The Relations of the Medical Profession to the Use and Abuse of Alcoholic Liquors," and did not seek any expression of opinion by formal action upon the part of that body. J. P. LOGAN, M. D.

